# FORTIFYING GERONTOLOGY PROGRAMS: HARNESSING COLLABORATIONS ACROSS CAMPUS

**DOI:** 10.1093/geroni/igae098.1620

**Published:** 2024-12-31

**Authors:** Tina Newsham, Elizabeth Fugate-Whitlock

**Affiliations:** University of North Carolina Wilmington, Wilmington, North Carolina, United States; University of North Carolina Wilmington, Wilmington, North Carolina, United States

## Abstract

Having joined the Age Friendly University Global Network and continuing the transition to an R2 institution, the University of North Carolina Wilmington is poised for strengthening its graduate program in gerontology. The program, like others, was once on the low productivity list and considered for dissolution, has only two full-time faculty members making collaborative efforts vital. To facilitate recruitment into the MS in Applied Gerontology and to situate the program firmly in the university’s infrastructure, we have initiated a variety of collaborative and interdisciplinary efforts. A set of five pathways known as “4 + 1” or “combined programs” allowing qualified undergraduate students to take a portion of their coursework at the graduate level. These students explore their interest in gerontology, become invested in gerontology education, and meet undergrad and graduate requirements simultaneously. Students are then able to complete their MS in Applied Gerontology in as little as one more year. In addition, new pathways have been created through combining teaching research, and scholarship endeavors. GRN faculty partner with colleagues (affiliated faculty) from Applied Health programs, Nursing, Social Work, Sociology/Criminology, Dance, and more to write grants, develop community engagement projects, and create meaningful opportunities for campus and community members to learn about gerontology. These efforts also provide opportunities to highlight careers in aging creating further interest in gerontology education. Such collaborations have created a steady stream of students into the graduate program, yet create their own challenges (as well as new opportunities).

